# Assessing activity limitations experienced by persons with rheumatoid arthritis to inform appropriate selection of patient-reported outcomes measures: a qualitative study

**DOI:** 10.1186/s41687-025-00955-5

**Published:** 2025-11-04

**Authors:** Heqin Yang, Asmita Priyadarshini Khatiwada, Jeffrey R. Curtis, W. Benjamin Nowell, Kelly Gavigan, Gary Hawkins, Cheryl Seals, Chad G. Rose, Nicholas P. McCormick, Kimberly B. Garza

**Affiliations:** 1 Department of Health Outcomes Research and Policy, Auburn University, Auburn, AL USA; 2 Division of Clinical Immunology and Rheumatology, University of Alabama at Birmingham, Birmingham, AL USA; 3 Global Healthy Living Foundation, Upper Nyack, NY USA; 4 Present Address: Regeneron Pharmaceuticals Inc., Sleepy Hollow, NY USA; 5 Innovation and Research Commons, Auburn University, Auburn, AL USA; 6 Department of Computer Science and Software Engineering, Auburn University, Auburn, AL USA; 7 Department of Mechanical Engineering, Auburn University, Auburn, AL USA

**Keywords:** Activities of daily living, Content validation, Patient reported outcomes measures, International classification of functioning, disability and health, Arthritis, rheumatoid

## Abstract

**Objectives:**

To identify daily activity limitations, including but not limited to impairments in physical function, experienced by persons with rheumatoid arthritis (RA) and identify Patient-Reported Outcomes Measurement Information System (PROMIS) short form (SF) scales that can measure these limitations.

**Methodology:**

A cross-sectional, web-based survey was conducted among a diverse group of adults with RA across the spectrum of disease activity. PROMIS Upper Extremity (UE) SF7a, Physical Function (PF) SF8b, and a Task Difficulty Scale were used to assess daily activity limitations experienced by persons with RA in the United States. An open-ended question asking what other daily activity limitations respondents experienced was also included. The daily activity limitation in the text response and the three scale items were deductively coded using the International Classification of Functioning, Disability, and Health (ICF) based on an established linking rule. Potential PROMIS SF scales were identified and linked to ICF to measure some activity limitations reported by persons with RA. PROMIS PF SF10a was linked to ICF and compared with UE SF7a and PF SF8b.

**Results:**

Eighty-three out of 99 RA patient respondents answered the open-ended question, the majority (> 70%) of whom had severe or moderate physical function limitations. All 9 second-level sub-categories of the Activities and Participation (A&P) in ICF were linked to the text response to the open-ended question. UE SF7a and PF SF8b were linked to four of these second-level sub-categories (e.g., Mobility), while additional four second-level sub-categories (e.g., General tasks and demands, Interpersonal interactions and relationships) linked to PROMIS Fatigue SF7a and Ability to Participate in Social Roles and Activities (APS) SF4a. Moreover, PF SF10a was linked to four of these second-level sub-categories too, but with fewer items than UE SF7a plus the PF SF8b. Some activity limitations, such as driving and using telecommunication devices, were not linked to any items of the five PROMIS SF scales.

**Conclusions:**

Persons with RA reported a variety of activity limitations across multiple domains, including physical function, telecommunication, social interactions, and other aspects of daily living, which could be a focus for goal-setting communication in the clinical setting. PROMIS PF SF10a may be a more effective and efficient scale to measure 4 sub-categories of daily activity limitations. To more comprehensively assess the spectrum of the impact of RA, it appears advisable to also use PROMIS Fatigue SF7a and APS SF4a to examine General tasks and demands, and Interpersonal interactions and relationships limitations.

**Supplementary Information:**

The online version contains supplementary material available at 10.1186/s41687-025-00955-5.

## Introduction

Rheumatoid arthritis (RA) is a systemic autoimmune condition associated with a chronic inflammatory process that affects around 0.3-1% of the world population [1]. The symptoms of RA vary significantly, with pain, stiffness, and fatigue being the most common, which affect daily life in physical, psychological, and social domains [1, 2]. If left untreated, RA can lead to loss of function, disability, an increased burden of disease, and decreased quality of life [3]. Remission is the desired target for treatment with immunomodulators [4]. While many biologics and other targeted therapies exist to reduce inflammation, many RA patients do not have treatment escalated to the optimized level to reach the desired state of remission, as recommended in national guidelines [4, 5]. When setting treatment targets (low disease activity or remission), clinicians should consider patient preferences, comorbidities, and other personal contexts, such as daily activity limitations, to meet patients’ needs [6]. A cross-sectional study showed that poor overall health status is positively associated with difficulty in executing activities of daily living (ADLs) among persons with RA, such as participating in leisure activities, attending social events, walking, and preparing meals [7]. Holistic care focusing on ADLs and clearly communicating how disease activity control benefits ADLs may support treatment escalation and improve patient outcomes [7, 8]. Therefore, better understanding of the daily activity limitations experienced by persons with RA is essential and could help set appropriate treatment targets for patients and encourage proper escalation of therapy.

Patient-reported outcome measures are widely used to capture the experience, behavior, and treatment outcome of patients, and to engage them as partners in their care [9]. In the Patient Reported Outcome Measurement Information System (PROMIS), physical function (PF) measures assess the ability to perform ADLs and some instrumental activities of daily living (IADLs) [10]. The PROMIS PF measures can be electronically administered via computerized adaptive tests (CAT) for greater efficiency and reliability, but paper-based short forms (SF) (e.g., the upper extremity (UE) SF7a and PF SF8b, 10a, and 20a) are also available for settings where CAT administration is not feasible [11–13]. PROMIS PF measures are designed to be generic but have been tested among RA patients [14–16]. However, differential item functioning (DIF) (i.e., the association between an item and its underlying construct may vary depending on the specific patient population) was detected in PROMIS PF items between cardiology and rheumatology patients, predominately from the UE subdomain [15]. With multiple PROMIS PF SF measures available and RA-related DIF, clinicians and researchers are left with uncertainty as to which SF measure(s) should be used to evaluate the domains of daily activity limitations among persons with RA sufficiently and efficiently.

The conceptual model and content validity, which originated from the targeted patient population and experts, are two important criteria to consider when selecting relevant and comprehensive patient-reported outcome measures [17]. Common activity limitations reported by persons with RA can inform clinicians and researchers about what should be measured. Our study aimed to (1) identify domains of daily activity limitations, including but not limited to impairments in physical function, experienced by persons with RA and (2) identify PROMIS SF scales that can measure these limitations by mapping the conceptual coverage. The identified daily activity limitation could be used in patient-physician communication to set appropriate treatment goals that align with the daily living situation of persons with RA. Further, these findings can inform clinicians’ selection of appropriate PROMIS SF measures to assess daily activity limitations, which could help encourage proper escalation of therapy.

## Methods

### Participants

A cross-sectional web-based survey was conducted among members of PatientSpot (PS; formerly ArthritisPower), an online patient-powered research network (registry) in the United States (U.S.) that includes adults with rheumatologic conditions [18]. Members of PS were invited to participate in this survey if they participated in a separate educational priorities survey, indicated they were willing to participate in a follow-up survey, and had a diagnosis of RA [19]. The educational priorities (primary) survey required that the participant be 19 years of age or older (based on the minimum age of consent which varies somewhat by state) and have a diagnosis of RA or another arthritis, inflammatory, musculoskeletal, or autoimmune condition, as diagnosed by a rheumatologist according to self-report in the patient registry. Only those self-reporting RA were invited to participate in this follow-up survey.

### Measurements

The survey included questions about demographics, time since RA diagnosis, scales that measure perceptions regarding the impact of RA on life in the past year [20], perceptions of current RA treatment, and validated scales (PROMIS SF Adult v2.0 – Physical Function SF8b [PF SF8b] [11] and Upper Extremity SF7a [UE SF7a]) [13] that measure physical function. This survey also included a task difficulty scale (TDS) with 5-point Likert-type scale items (*n* = 13) (Appendix 1) to assess participants’ self-reported difficulty performing daily activities not specifically included in the UE SF7a or PF SF8b. The TDS was developed by the authors and informed by a list of common daily activity limitations among persons with RA published by the Centers for Disease Control and Prevention [21, 22] and the major domains of the IADLs [23] and ADLs [24] (Appendix 1). An open-ended text response item asked participants to describe “other barriers you have experienced in performing everyday activities due to your rheumatoid arthritis that are not included above” (i.e., activities were not captured by the task difficulty scale nor either of the PROMIS PF SF8b or UE SF7a).

### Data analysis

A descriptive analysis was conducted to characterize the sample. T-scores of PROMIS PF SF8b and UE SF7a were generated based on scoring instructions [25]. Based on general guidelines for interpreting PROMIS scores, the severity levels of physical function are categorized as “Within normal limits, T-score ≥ 45.1”, “Mild, 40.1 ≤ T-score ≤ 45”, “Moderate, 30.1 ≤ T-score ≤ 40,” and “Severe, T-score ≤ 30” [26].

**Fig. 1:**
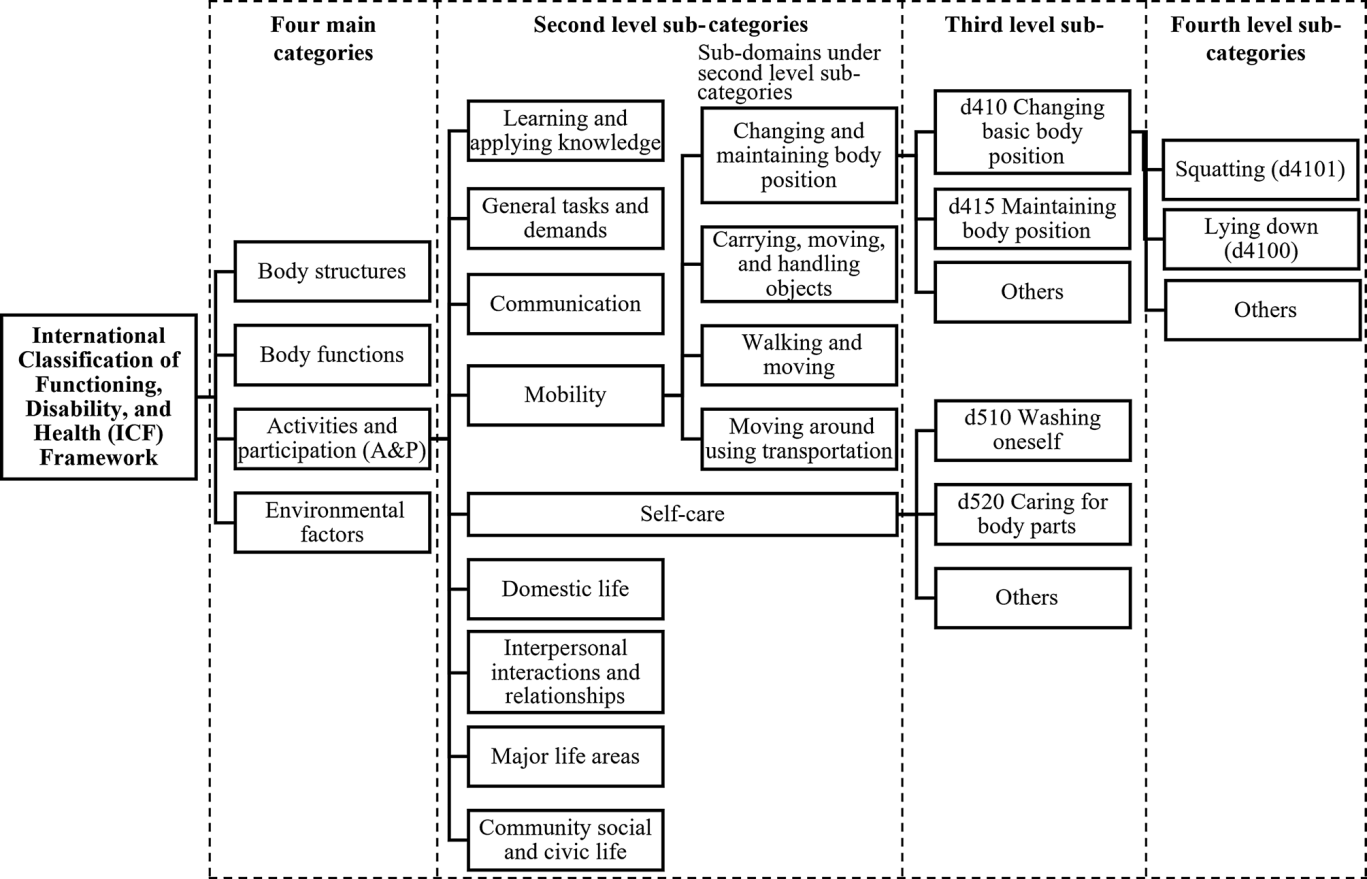
Diagram of International Classification of Functioning, Disability, and Health (ICF). This figure was created based on the International Classification of Functioning, Disability and Health (ICF) framework available on the World Health Organization’s website: https://icd.who.int/dev11/l-icf/en#/http%3a%2f%2fid.who.int%2ficd%2fentity%2f464886707

The International Classification of Functioning, Disability, and Health (ICF), which is widely used to understand the health and functioning experience of people with chronic conditions [27, 28]. It provides a comprehensive conceptual framework to summarize and describe the domains of activity limitation experienced by persons living with RA. Endorsed in 2001 by the World Health Assembly, the ICF serves as a universal framework and classification system to describe and classify health, functioning, and disability [27]. Deductive coding of the text responses to the open-ended survey item (text response in short) was conducted using the framework of the ICF [29]. The ICF framework (Fig. 1) has four main mutually exclusive categories of functioning and disability: Body structures, Body functions, Activities and Participation (A&P), and Environmental factors [29]. Under the four main categories of ICF, there are second- (e.g., Changing and maintaining body position), third- (e.g., Changing basic body position [d410]), and fourth-level (e.g., Lying down [d4100]) sub-categories to provide vocabularies to describe individual health conditions and patients’ lived experience of health. Higher levels of sub-categories (with the fourth-level being the highest) represent increasingly more detailed, specified health information [29].

This study only used the sub-categories belonging to the main category A&P to map to daily activity limitations experienced by persons with RA. The second-level sub-categories of A&P include (1) Learning and applying knowledge, (2) General tasks and demands, (3) Communication, (4) Mobility, (5) Self-care, (6) Domestic life, (7) Interpersonal interactions and relationships, (8) Major life areas, and (9) Community social and civic life [30]. As to the second-level sub-categories, some of them are further grouped into sub-domains, such as Mobility, which include Changing and maintaining body position and others. Then, these sub-domains under the second-level sub-categories, such as the subdomain of Changing and maintaining body position was divided into the third-level sub-categories (ranging from d410-d499), namely Changing basic body position (d410), Maintaining body position (d415), etc. In addition, some second-level sub-categories were directly divided into third-level sub-categories, for example, Self-care was divided into Washing oneself (d510), Caring for body parts (d520), and others ranging from d530 to d599. Lastly, the third-level sub-categories were divided into the fourth-level sub-categories, for example, Changing basic body position (d410) was divided into Lying down (d4100), Squatting (d4101), and others ranging from d4103-d4759 (Fig. 1) [30].

Each concept from the participants’ text responses was linked to the most appropriate higher level ICF sub-category according to established linking rules [29]. Most concepts from the data were linked to fourth-level sub-categories; when no fourth-level sub-categories could be matched to a concept, it was subsequently linked to a third-level sub-category such as Changing and maintaining body position, other specified and unspecified (others) (d429). The concepts from items of the three scales measuring activity limitations (i.e., PROMIS UE SF7a, PF SF8b, and the TDS) were linked to appropriate ICF sub-categories by following the linking rule. The concepts from the text responses and items from the three scales were compared to show which concepts could be measured by these scales. In addition to the category classification, the ICF framework specifies qualifiers to segregate the extent of functioning. However, the ICF qualifier was not used to code the text response and the response options for PROMIS measures because it is arbitrary to determine the severity of daily activity limitations reported in the text responses in this study.

The available PROMIS SF measures were reviewed to identify other potential SF scales that could measure the participants reported daily activity limitations but not linked to PROMIS UE SF7a or PF SF8b. Consequently, the items of PROMIS Fatigue SF7a and Ability to Participate in Social Roles and Activities (APS) SF4a were linked to the ICF using the same linking rules. Moreover, clinical practice guidelines recommend the use of the PROMIS Physical Function-Short Form 10a (PF SF10a) to measure physical function among RA patients in routine clinical practice [31], subsequently informing its inclusion in the coding procedure. The second- and third-level sub-categories of A&P linked to PROMIS PF SF10a and the two implemented PROMIS SF scales (UE SF7a and PF SF8b) in this study were compared.

Two coders (HY and APK) independently coded the qualitative data from the participants’ text responses to ensure study dependability and trustworthiness [32]. Following independent linking of text response to the ICF framework, the two coders met to compare and discuss their codes to affirm inter-coder consistency. In instances wherein the two coders utilized differing fourth-level sub-categories, which fall into the same third-level sub-categories, they were treated as agreement. This is evident in cases such as Putting on clothes (d5400) being identified by HY versus Other specified dressing: buttoning (d5408) being identified by APK for persons with RA reporting “I’ve had to have coworkers help button some of my shirts.” Following discussion, the two coders made changes to their codes to inform future coding practices and consistency. Next, the fourth-level categories of daily activity limitations identified in the text response among all participants were summarized after modification and the two researchers met again to discuss the inclusion of fourth-level categories to reach consensus. Discrepancy between the two initial coders was addressed by a third independent coder (KBG). The third-level sub-categories of the ICF framework were used to determine if the PROMIS SF scales can be used to measure the concepts from the text response, as fourth-level sub-categories provide too detailed information, which may not be appropriate for use in the clinical setting.

## Result

### Characteristics of participants

A total of 99 adults with RA completed the survey. The majority of participants were between 45 and 64 years old (61.3%), female (84.9%), White (92.5%), married (50.5%), hold a four-year college or higher degree (57.0%), had an annual household income of $50,000 or more (49.5%), and diagnosed with RA for more than 5 years (69.7%) (Table 1).

**Table 1:** Characteristics of rheumatoid arthritis (RA) participants

Characteristics	*N* = 99^1*^
Age Group	
Less than 45 years	5 (5.4%)
45–64 years	57 (61.3%)
65 years and older	31 (33.3%)
Gender	
Male	13 (14.0%)
Female	79 (84.9%)
Other	1 (1.1%)
Race	
White	86 (92.5%)
Non-White	5 (5.4%)
Prefer not to say	2 (2.2%)
Marital Status	
Married	47 (50.5%)
Unmarried	43 (46.2%)
Prefer not to say	3 (3.2%)
Education	
Less than 4 year degree	40 (43.0%)
At least a 4 year college degree	53 (57.0%)
Annual Household Income	
Less than $50,000	36 (38.7%)
$50,000 and more	46 (49.5%)
Prefer not to say	11 (11.8%)
RA Disease Duration	
Not more than 5 years ago	30 (30.3%)
Diagnosis > 5 years ago	69 (69.7%)

^1^n (%)

*93 out of 99 respondents answered the demographic characteristics section

Based on the T-score of upper extremity short form 7a (UE SF7a), most participants had severe (*n* = 26 [28.0%]) or moderate (*n* = 47 [50.5%]) limitation with upper extremity function, while few participants had mild limitation (*n* = 7 [7.5%]) or within normal limits (*n* = 13 [14.0%]) (Table 2). The T-scores of physical function short form 8b (PF SF8b) showed similar severity categorization but with more participants (*n* = 22 [23.2%]) having mild limitation of physical function.

**Table 2:** Severity of physical function limitations based on PROMIS short form T-scores

Measures	Mean (SD)	Severity Level	*n* (%)
Upper Extremity short form 7a (T-score)	34.85 (7.55)	Severe	26 (28.0)
		Moderate	47 (50.5)
		Mild	7 (7.5)
		Within normal limits	13 (14.0)
Physical Function short form 8b (T-score)	37.30 (5.62)	Severe	6 (6.3)
		Moderate	61 (64.2)
		Mild	22 (23.2)
		Within normal limits	6 (6.3)

Severe: T-score ≤ 30; Moderate: 30.1 ≤ T-score ≤ 40; Mild: 40.1 ≤ T-score ≤ 45; Within normal limits: T-score ≥ 45.1

### Sub-categories of activity and participation (A&P) linking to the text response

**Fig. 2:**
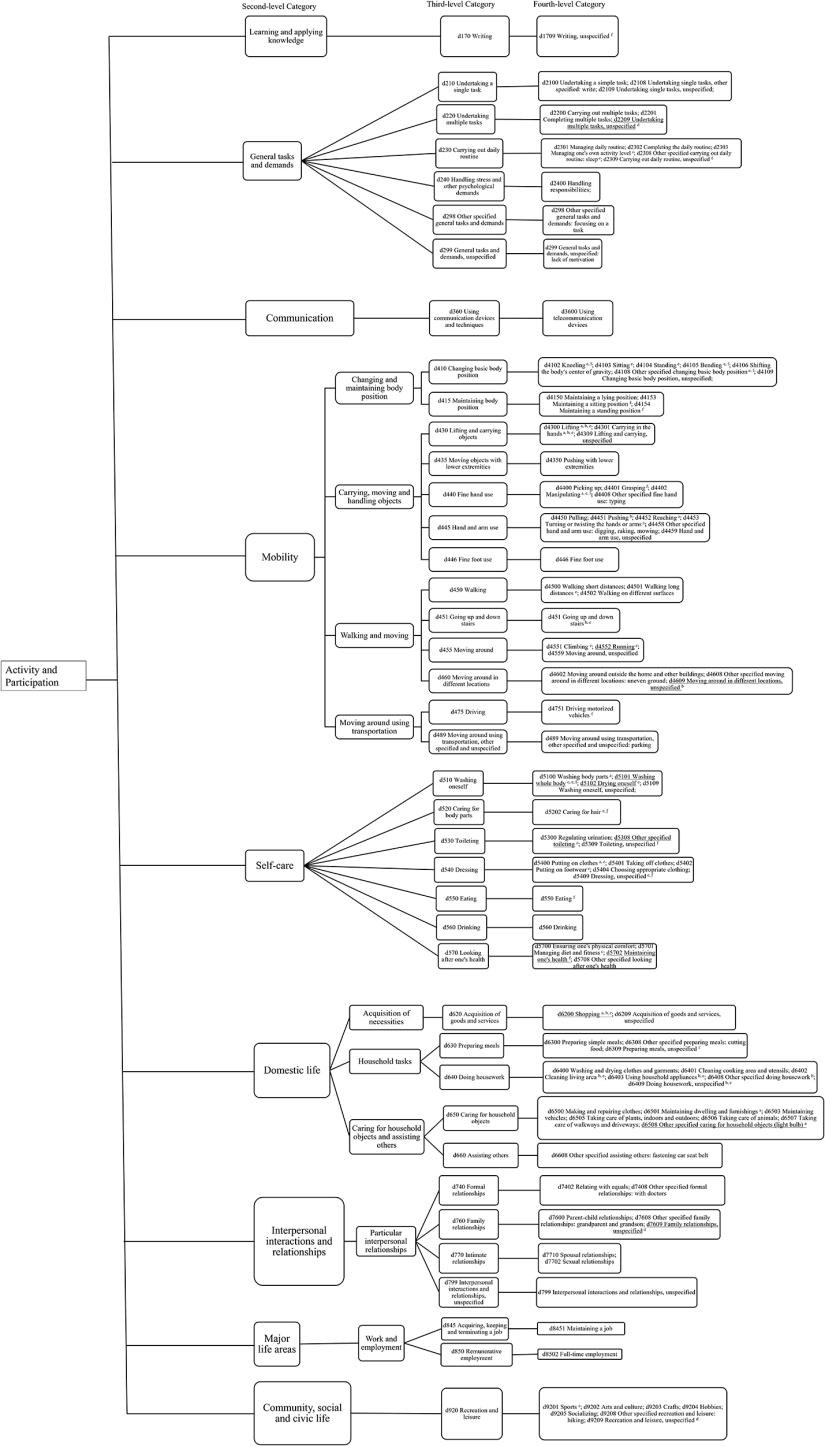
The sub-categories under activity and participation in ICF linked to the text response to the open-ended question and PROMIS short form measures. The sub-categories with ^a, b,c, d,e^ mean that they were linked to the items in PROMIS short form measures. ^a^ Upper Extremity SF 7a; ^b^ Physical Function SF 8b; ^c^ Fatigue SF 7a; ^d^ Ability to Participate in Social Roles and Activities - SF 4a; ^e^ Physical Function – Short Form 10a. The sub-categories with ^f^ mean that they were linked to the items in the task difficulty scale. _ The sub-category with underline means it was not linked to the text response to the open-ended question. ICF: International Classification of Functioning, Disability and Health; PROMIS: Patient Reported Outcomes Measurement Information System

The open-ended question asked what other activity limitations they had after three scales (i.e., PROMIS UE SF7a, PF SF8b, and the TDS) were displayed. Eighty-three (84%) participants responded to the open-ended question. The non-respondents (*n* = 16) were similar to respondents in terms of RA severity and demographic characteristics. Four of the 83 respondents (4.82%) reported there was no further daily activity limitation. The text responses were linked to forty unique third-level sub-categories under A&P in ICF, such as Undertaking a single task (d210), Carrying out daily routine (d230), Handling stress and other psychological demands (d240) (Fig. 2). The fourth-level sub-categories directly linking to the text response are also shown in Fig. 2. Different portions of third-level sub-categories under all 9 second-level sub-categories of A&P linked to the text response (Learning and applying knowledge [1 out of 26], General tasks and demands [6 out of 6], Communication [1 out of 18], Mobility [13 out of 22], Self-care [7 out of 9], Domestic life [5 out of 11], Interpersonal interactions and relationships [4 out of 11], Major life areas [2 out of 18], and Community social and civic life [1 out of 7]).

### Items of three scales (i.e., PROMIS UE SF7a, PF SF8b, and the TDS) linking to sub-categories of the A&P

In Table 3, the “+” symbol indicates that the scale is linked to the third-level sub-category of activity limitation listed in that row. When multiple scales had “+” in the same row, it suggests that they cover the same activity limitation. If only one “+” appears in a row, it indicates that the activity limitation is measured by that single scale. Rows without any “+” denote activity limitations not captured by any of the listed scales.

**Table 3:** Comparison of sub-categories under activity and participation (A&P) in ICF linking to the text response to the open-ended questions and items in the PROMIS short form measures

Second-level sub-categories linked to text response	Third-level sub-categories linked to text response	ICF A&P core sets for rheumatoid arthritis^#^	Upper Extremity SF 7a	Physical Function SF 8b	Task Difficulty scale	Fatigue SF 7a	Ability to Participate in Social Roles and Activities - SF 4a	Physical Function – SF 10a	HAQ
Learning and applying knowledge	d163 Thinking *	+				+			
	d170 Writing				+				
General tasks and demands	d210 Undertaking a single task								
	d220 Undertaking multiple tasks						+		
	d230 Carrying out daily routine	+				+	+		
	d240 Handling stress and other psychological demands								
	d298 Other specified general tasks and demands								
	d299 General tasks and demands, unspecified								
Communication	d360 Using communication devices and techniques								
Mobility-Changing and maintaining body position	d410 Changing basic body position	+			+			+	+
	415 Maintaining body position	+			+				
Mobility-Carrying, moving and handling objects	d430 Lifting and carrying objects	+	+	+				+	+
	d435 Moving objects with lower extremities								
	d440 Fine hand use	+	+		+			+	+
	d445 Hand and arm use	+	+	+					+
	d446 Fine foot use								
Mobility-Walking and moving	d450 Walking	+						+	+
	d451 Going up and down stairs			+				+	
	d455 Moving around	+						+	+
	d460 Moving around in different locations	+		+					
Mobility-Moving around using transportation	d475 Driving	+			+				
	d489 Moving around using transportation, other specified and unspecified								
Self-care	d510 Washing oneself	+	+		+	+		+	+
	d520 Caring for body parts	+			+			+	+
	d530 Toileting	+			+			+	+
	d540 Dressing	+	+		+			+	+
	d550 Eating	+			+				+
	d560 Drinking	+							+
	d570 Looking after one's health	+			+	+			
Domestic life-Acquisition of necessities	d620 Acquisition of goods and services	+	+	+				+	+
Domestic life- Household tasks	d630 Preparing meals	+			+				
	d640 Doing housework	+		+				+	+
	d649 Household tasks, other specified and unspecified*	+		+		+			
Domestic life-Caring for household objects and assisting others	d650 Caring for household objects		+						
	d660 Assisting others	+							
Interpersonal interactions and relationships-Particular interpersonal relationships	d710 Basic interpersonal interactions*	+					+		
	d740 Formal relationships	+							
	d750 Informal social relationships*	+					+		
	d760 Family relationships	+					+		
	d770 Intimate relationships	+							
	d799 Interpersonal interactions and relationships, unspecified								
Major life areas-Work and employment	d845 Acquiring, keeping and terminating a job								
	d850 Remunerative employment	+							
	d859 Work and employment, other specified and unspecified*			+		+	+		
Community social and civic life	d920 Recreation and leisure	+					+	+	

* The third-level sub-categories were not linked to the text response to the open-ended question but linked to the PROMIS short form measures

# A previous study reported ICF core sets for rheumatoid arthritis [33]. The third-level categories of activity limitation included in ICF core sets but not identified in this study are: Carrying, moving, and handling objects, other specified and unspecified (d449); Moving around using equipment (d465); Using transportation (d470); Work and employment, other specified and unspecified (d859); Community life (d910)

ICF: International Classification of Functioning, Disability and Health; PROMIS: Patient Reported Outcomes Measurement Information System; HAQ: Health Assessment Questionnaire

+ The third-level sub-categories were linked to the items in the PROMIS short form measures

The items of PROMIS upper extremity (UE) SF7a and the physical function (PF) SF8b linked to 7 and 8 third-level sub-categories of A&P respectively (Table 3). The two scales linked to 12 third-level sub-categories in total because they linked to three same third-level sub-categories. Despite the open-ended question being displayed after the three scales (i.e., PROMIS UE SF7a, PF SF8b, and the TDS), all the third-level sub-categories of A&P linking to UE SF7a were linked to the text response reported by persons with RA. Two of the 8 third-level sub-categories linking to the PF SF8b had similar, but not exact third-level sub-categories of A&P linked to the text response. Four second-level sub-categories linking to the daily activity limitations in the text response were linked to either UE SF7a or PF SF8b, i.e., Mobility, Self-care, Domestic life, and Major life areas -Work and employment. Five second-level sub-categories linking to daily activity limitations in the text response were not linked to the two scales, i.e., Learning and applying knowledge, General tasks and demands, Communication, Interpersonal interactions and relationships, and Community and social and civic life.

The TDS linked to 12 third-level sub-categories of A&P, all of which were linked to the text response. This scale covered four second-level sub-categories in A&P. Moreover, 6 out of 7 third-level sub-categories under Self-care were linked to this scale. Further, this scale linked to nine third-level sub-categories of A&P that were not linked to PROMIS UE SF7a or PF SF8b.

### Sub-categories of A&P linking to text response that were not linked to UE SF7a and PF SF8b

PROMIS short forms were reviewed to identify potential short form scales to link to the five second-level sub-categories which were linked to the participants’ text responses but not linked to UE SF7a or PF SF8b, resulting in including PROMIS Fatigue SF7a and Ability to Participate in Social Roles and Activities (APS) SF4a in the coding procedure (Table 3). Fatigue SF7a linked to 6 third-level sub-categories under the A&P, which were under five second-level sub-categories. Three of the 6 third-level sub-categories were linked to the daily activity limitations reported in the text response. The APS SF4a was linked to 7 third-level sub-categories under the A&P in ICF, which were under four second-level sub-categories. Four of the 7 third-level sub-categories were linked to the daily activity limitations reported in the text response.

### Comparing the sub-categories of A&P linking to PROMIS PF SF10a with UE SF7a plus PF SF8b

PROMIS PF SF10a linked to 13 third-level sub-categories of A&P, all of which belonged to the 40 daily activity limitations self-reported by participants in the text response (Table 3). The 13 third-level sub-categories were under four second-level sub-categories which linked to the text response and left five second-level sub-categories not linking to PF SF10a. PF SF10a linked to the third-level sub-category of Recreation and leisure (d920), belonging to the second-level sub-category of Community social and civic life, which was not linked to UE SF7a or the PF SF8b. Three third-level sub-categories were linked to UE SF7a or the PF SF8b, but not PF SF10a. Worth noting, none of the five PROMIS short form measures linked to the sub-categories in Mobility-Moving around using transportation, and Communication - specifically using telecommunication device. An figure is also provided to visualize the overlapping, distinct coverage, and uncovered second- and third-level sub-categories of daily activity limitations (Appendix 2).

## Discussion

This study aimed to identify the activity limitations experienced by persons with RA and the appropriate PROMIS SF scales to adequately measure these activity limitations, informed both by free text responses from RA patients and using a well-established ICF framework as a method to organize relevant concepts. Using free text responses to an open-ended question, persons with RA reported 40 unique third-level sub-categories in all 9 s-level sub-categories of A&P of daily activity limitations. These limitations encompassed not only physical function, but also social interaction, telecommunication, and other aspects of daily living. The PROMIS upper extremity (UE) SF7a and the physical function (PF) SF8b were linked to 12 third-level under 4 out of the 9 second-level sub-categories, which means the two scales can be used to measure the daily activity limitations in these domains. The items in PROMIS Fatigue SF7a and APS SF4a linked to 4 of the remaining 5 second-level sub-categories and could be used to measure these daily activity limitations experienced by persons with RA. Moreover, items in PF SF10a were linked to 4 second-level sub-categories of daily activity limitations reported by persons with RA but linked to 13 third-level sub-categories of daily activity limitations, which was one more than UE SF7a plus PF SF8b. None of the five PROMIS SF scales linked to the sub-domain of the second-level sub-category Mobility-Moving around using transportation and Communication, under which, limitations in driving and using telecommunication devices were reported by persons with RA.

The daily activity limitations experienced by persons with RA in this study overlapped the majority of the comprehensive ICF core sets for RA, which were generated from a systematic review, empirical data collection, and expert consensus over 20 years ago [33]. The comprehensive ICF core set included 32 third-level sub-categories in the main category of A&P in the ICF (Table 3). As mentioned above, our study found 40 third-level sub-categories of A&P in text response to the daily activity limitation question. The daily activity limitations reported by persons with RA in this study overlapped 27 out of 32 third-level sub-categories in the RA ICF core set, which suggests our finding provided a comprehensive coverage of relevant limitations. The specific third-level categories from the RA ICF Core Set that were not identified in this study are listed in Table 3. In addition, we identified several third-level sub-categories of activity limitation that were not included in the RA ICF core set.

PROMIS Fatigue SF7a and APS SF4a can be used to measure daily activity limitations experienced by persons with RA which were not covered by PROMIS PF SF10a or UE SF7a plus PF SF8b. A previous study reported that RA patients ranked PROMIS Fatigue as the most important assessment they want to use to monitor their symptoms [34]. Our study found that PROMIS Fatigue SF7a can be used to measure four second-level sub-categories of the activity limitations experienced by persons with RA, which is consistent with their result. It is worth noting that some items in the Fatigue SF7a focus solely on energy levels - such as ‘How often did you feel tired?’ - without directly addressing daily activity. This makes the scale less appropriate when daily activity limitations are not primarily caused by fatigue.

The daily activity limitations of social participation reported in this study align with prior studies that RA patients often struggle with social activities [35] and experience high levels of loneliness [36]. Assessing social participation may help better understand their daily activity limitations and overall well-being [37]. For the measures in the PROMIS social health domain, there are short-form scales focusing on companionship, emotional support, instrumental support, satisfaction with participation in discretionary social activities, social isolation, and others. The items in these scales examine the perceived availability of support from others, the satisfaction of personal physical ability, and the feeling of respondents, but not the perceived ability to participate in activity [38]. Thus, the ability to participate in social roles and activities scale (APS) was chosen to measure daily activity limitations of social participation. Though PROMIS APS SF6a and SF8a are also available, this study found APS SF4a, with 4 items, linked to four second-level sub-categories in A&P. Moreover, three out of the four second-level sub-categories were not linked to PROMIS UE 7a or PF 8b, which made it a valid option to assess daily activity limitations in social participation among persons with RA.

The PROMIS PF SF10a was linked to the daily activity limitations experienced by persons with RA in four second-level sub-categories (i.e., Mobility, Self-care, Domestic life, and Community social and civic life) in the main domain of A&P of ICF. It linked to more third-level sub-categories in A&P than both UE SF7a and PF SF8b. Thus, PF SF10a can measure more domains of activity limitation with fewer items, which suggested that PF SF10a was a more efficient and effective tool to measure the daily activity limitations experienced by persons with RA. Moreover, a previous study found HAQ-DI and 36-item Short Form Health Survey (SF-36) physical functioning scale (10 items) linked to 14 and 8 ICF RA core sets (as in third-level sub-category), respectively [39]. HAQ-DI and SF-36 physical functioning scale linked to one fewer second-level sub-categories than PROMIS PF SF10 ^39^. This suggested that PROMIS PF SF10a was slightly more comprehensive than HAQ-DI and SF-36 physical functioning scale to measure the daily activity limitations experienced by persons with RA. Though the PROMIS PF SF10a linked to more second-level subcategories of activity limitation reported by persons with RA, these scales (i.e., PROMIS PF SF10a, UE SF7a, PF SF8b, and HAQ-DI; Table 3) linked to some different third-level sub-categories of activity limitation. For example, moving around in different locations (d460) was only linked to PROMIS PF SF8b and Caring for household objects (d650) was only linked to PROMIS UE SF7a. Scales other than PROMIS PF SF10a should be considered if the activity limitations, like moving around in different locations, were the intended concepts to measure.

It is worth noting that some activity limitations reported by persons with RA were not linked to any of the validated scales, such as Driving (d475), which was linked to the task difficulty scale (TDS) in this study. Future research could validate the scale measuring driving, which was an activity limitation identified in this study and included in the ICF RA core set. Moreover, in this study, persons with RA reported activity limitation in Using telecommunication device and technique (d360) (such as texting, typing, phone constantly dropping), which could be an important daily activity among persons with RA residing in the U.S. with 97% of Americans using a mobile phone [40]. Previous reviews demonstrate that the use of mobile health tools increased and was proved to promote reaching rapid remission and improve patient-physician interaction by engaging and activating patients in management of RA [41, 42]. However, the activity limitation related to using a telecommunication device could inhibit persons with RA from adopting and maintaining the use of phone-based interventions. Furthermore, high intensity use of smartphones diminishes hand grip strengths and hand function leading to decreased hand grip strength even among the healthy population [43]. Future research could examine the extent of limitation in using telecommunication devices and the importance of this activity limitation among persons with RA. This activity limitation might be important to consider and measure when designing and implementing phone-based interventions.

### Limitations

This study has several limitations. The daily activity limitations were reported by persons with RA who were members of an online patient-powered research network (registry) in the U.S. and this study did not focus on comorbidities of participants. Despite the fact that third-level sub-categories of daily activity limitations largely overlapped with the result of previous expert consensus [33], clinicians should be cautious about generalizing the result to RA patient populations with different demographic profiles, socioeconomic backgrounds, or specific comorbid conditions (e.g. chronic lung disease, diabetes). For example, participants in this study were primarily white and female, with high levels of education and high annual income, and capable of reading and responding to the survey so they may not represent individuals with cognitive impairment or low literacy. In addition, the identified daily activity limitations were based on a single open-ended question in an online survey that was administered in a single wave. We did not have the opportunity to clarify the meaning of the response or elicit more information to better understand the daily activity limitations experienced by persons with RA. For instance, participants reported unable to or difficult to write without clarifying if they can produce symbols or language to convey information. The activity limitation about applying knowledge remained unclear in this study. Accordingly, we may have missed some relevant activity limitations that are meaningful to patients. Future studies with multiple waves of data collection that prompt patients until thematic saturation is reached, and oversampling for patient subgroups that may be under-represented in this cohort, could enhance convergence and ensure a more comprehensive understanding of patient-relevant limitations. The question was worded as “other barriers you have experienced in performing everyday activities due to your rheumatoid arthritis that are not included above”; therefore, respondents may not have included daily activity limitations that had already been assessed by the PROMIS UE7a, PROMIS PF8b, and the task difficulty scale. However, participants in this study reported specific daily activity limitations other than the ones in the measurements, which linked the same third-level sub-categories of A&P linking to these measures. Only one third-level sub-category of A&P (i.e., Work and employment, others [d859]) linked to PROMIS PF8b but not the text response to the open-ended question. However, the respondents reported specific work and employment related daily activity limitations, such as Full-time employment (d8502). Moreover, this study did not assess the severity of the daily activity limitations, which could impose different influence on patients’ life and treatment needs. Future research can include the severity of daily activity limitations and use the ICF qualifier to categorize them [30].

### Implication

This study summarized daily activity limitations experienced by persons with RA, which could inform the communication between physicians and RA patients to engage patients in medical decisions and set treatment goals. The terms of activity limitation followed the ICF framework, which provides an organizational hierarchy to understand the activity limitation among persons with RA. Clinicians could refer to these terms to elicit daily activity limitations and tailor treatment to individual patients with RA. Further, this study confirmed the American College of Rheumatology’s recommendation of using the PROMIS SF10a to examine RA patients’ daily activity limitations when the PROMIS PF CAT is not feasible to implement [31]. Clinicians can use PROMIS PF SF10a if they are considering measuring the activity limitations of RA patients and working with patients to address those issues. In addition, clinicians could consider using PROMIS Fatigue SF7a and APS SF4a when RA patients report potential activity limitations in the domains of General tasks and demands and Interpersonal interactions and relationships. Finally, the linking between PROMIS SF scales and the ICF framework guides researchers to decide which short-form scale to use for specific daily activity limitations among persons with RA.

## Conclusion

This study identified 40 unique daily activity limitations reported by persons with RA, encompassing a wide range of domains, including physical function, telecommunication, social interactions, and other aspects of daily living. In general, the PROMIS PF SF10a was a more effective and efficient tool for measuring daily activity limitations in the domains of Mobility, Self-care, Domestic Life, and Community, Social, and Civic Life among individuals with RA, compared to the combined use of UE SF7a and PF SF8b. However, certain specific concepts were only captured by the latter two scales. The daily activity limitations in General tasks and demands and Interpersonal interactions and relationships, which were not covered by PROMIS PF SF10a, could be measured by PROMIS Fatigue SF7a and APS SF4a among this population in clinical settings. Future research could examine the extent of limitations in using telecommunication devices and driving among persons with RA.

## Supplementary Information

Below is the link to the electronic supplementary material.

Supplementary Material 1

## Data Availability

The datasets used and/or analyzed during the current study are available from the corresponding author on reasonable request.

## Appendix 1. Items in Task Difficulty Scale

**Table:**

Task difficulty scale (Without any difficulty = 1; With a little difficulty = 2; With some difficulty = 3; With much difficulty = 4; Unable to do = 5)	Are you able to grasp small objects, such as an ink pen?
	Are you able to open a jar or pill bottle?
	Are you able to sit for about 2 h?
	Are you able to stand for about 2 h?
	Are you able to stoop, bend, or kneel?
	Are you able to drive short distances?
	Are you able to fasten your seat belt?
	Are you able to prepare dinner?
	Are you able to brush or comb your hair?
	Are you able to dress yourself?
	Are you able to bathe yourself?
	Are you able to feed yourself?
	Are you able to go to the bathroom by yourself?

## Appendix 2. PROMIS short forms and task difficulty scale linked to text responses: overlap, distinct coverage, and uncaptured activity limitations

(The figure is in a different file)

**(1) Learning and applying knowledge**

- d163 Thinking*
- d170 Writing

**(2) General tasks and demands**

- d210 Undertaking a single task
- d220 Undertaking multiple tasks
- d230 Carrying out daily routine
- d240 Handling stress and other psychological demands
- d298 Other specified general tasks and demands
- d299 General tasks and demands, unspecified

**(3) Communication**

- d360 Using communication devices and techniques

**(4) Mobility-Changing and maintaining body position**

- d410 Changing basic body position
- 415 Maintaining body position

**Mobility-Carrying, moving and handling objects**

- d430 Lifting and carrying objects
- d435 Moving objects with lower extremities
- d440 Fine hand use
- d445 Hand and arm use
- d446 Fine foot use

**Mobility-Walking and moving**

- d450 Walking
- d451 Going up and down stairs
- d455 Moving around
- d460 Moving around in different locations

**Mobility-Moving around using transportation**

- d475 Driving
- d489 Moving around using transportation, other specified and unspecified

**(5) Self-care**

- d510 Washing oneself
- d520 Caring for body parts
- d530 Toileting
- d540 Dressing
- d550 Eating
- d560 Drinking
- d570 Looking after one’s health

**(6) Domestic life-Acquisition of necessities**

- d620 Acquisition of goods and services

**Domestic life- household tasks**

- d630 Preparing meals
- d640 Doing housework
- d649 Household tasks, other specified and unspecified*

**Domestic life-Caring for household objects and assisting others**

- d650 Caring for household objects
- d660 Assisting others

**(7) interpersonal interactions and relationships-**Particular interpersonal relationships

- d710 Basic interpersonal interactions*
- d740 Formal relationships
- d750 Informal social relationships*
- d760 Family relationships
- d770 Intimate relationships
- d799 Interpersonal interactions and relationships, unspecified

**(8) Major life areas-Work and employment**

- d845 Acquiring, keeping and terminating a job
- d850 Remunerative employment
- d859 Work and employment, other specified and unspecified*

**(9) Community social and civic life**

- d920 Recreation and leisure

* These third-level subcategories were not linked to the text response to the open-ended question but linked to the PROMIS short form measures.

## Abbreviations

RA: Rheumatoid Arthritis
PROMIS: Patient-Reported Outcomes Measurement Information System
SF: Short Form
UE: Upper Extremity
PF: Physical Function
ICF: International Classification of Functioning, Disability, and Health
A&P: Activities and Participation
APS: Ability to Participate in Social Roles and Activities
TDS: Task Difficulty Scale
DIF: Differential Item Functioning
CAT: Computerized Adaptive Tests
ADLs: Activities of Daily Living
IADLs: Instrumental Activities of Daily Living
HAQ-DI: Health Assessment Questionnaire Disability Index
AIMS2: Arthritis Impact Measurement Scales 2
SF-36: The Short Form Health Survey
PS: PatientSpot
