# Supplementary material for: Assessing activity limitations experienced by persons with rheumatoid arthritis to inform appropriate selection of patient-reported outcomes measures: a qualitative study
Source: J Patient Rep Outcomes. 2025 Nov 4;9:129. doi: 10.1186/s41687-025-00955-5 (PMC12586814; doi:10.1186/s41687-025-00955-5)
Supplement: Supplementary file 1 — Supplementary Material 1 [file 41687_2025_955_MOESM1_ESM.docx]

**Appendix 2.** PROMIS Short Forms and Task Difficulty Scale Linked to Text Responses: Overlap, Distinct Coverage, and Uncaptured Activity Limitations


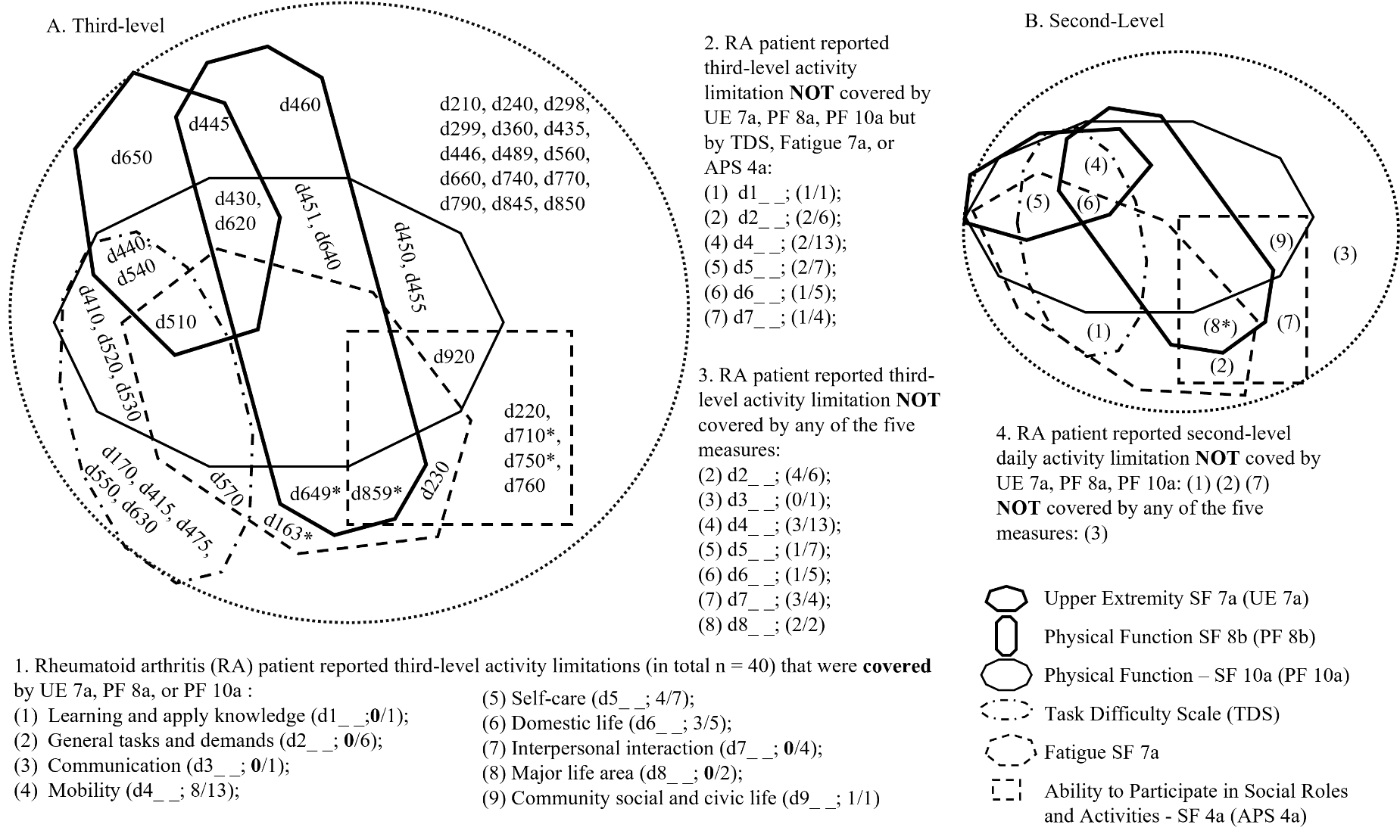


**(1) Learning and applying knowledge**

d163 Thinking*

d170 Writing

**(2) General tasks and demands**

d210 Undertaking a single task

d220 Undertaking multiple tasks

d230 Carrying out daily routine

d240 Handling stress and other psychological demands

d298 Other specified general tasks and demands

d299 General tasks and demands, unspecified

**(3) Communication**

d360 Using communication devices and techniques

**(4) Mobility-Changing and maintaining body position**

d410 Changing basic body position

415 Maintaining body position

**Mobility-Carrying, moving and handling objects**

d430 Lifting and carrying objects

d435 Moving objects with lower extremities

d440 Fine hand use

d445 Hand and arm use

d446 Fine foot use

**Mobility-Walking and moving**

d450 Walking

d451 Going up and down stairs

d455 Moving around

d460 Moving around in different locations

**Mobility-Moving around using transportation**

d475 Driving

d489 Moving around using transportation, other specified and unspecified

**(5) Self-care**

d510 Washing oneself

d520 Caring for body parts

d530 Toileting

d540 Dressing

d550 Eating

d560 Drinking

d570 Looking after one's health

**(6) Domestic life-Acquisition of necessities**

d620 Acquisition of goods and services

**Domestic life- Household tasks**

d630 Preparing meals

d640 Doing housework

d649 Household tasks, other specified and unspecified*

**Domestic life-Caring for household objects and assisting others**

d650 Caring for household objects

d660 Assisting others

**(7) Interpersonal interactions and relationships-**Particular interpersonal relationships

d710 Basic interpersonal interactions*

d740 Formal relationships

d750 Informal social relationships*

d760 Family relationships

d770 Intimate relationships

d799 Interpersonal interactions and relationships, unspecified

**(8) Major life areas-Work and employment**

d845 Acquiring, keeping and terminating a job

d850 Remunerative employment

d859 Work and employment, other specified and unspecified*

**(9) Community social and civic life**

d920 Recreation and leisure

* These third-level subcategories were not linked to the text response to the open-ended question but linked to the PROMIS short form measures
